# Predictive analysis of catecholamines and electrolytes for recurrence of orthostatic intolerance in children

**DOI:** 10.3389/fped.2023.1220990

**Published:** 2023-08-29

**Authors:** Lujie Chang, Lu Peng, Jianglin Liu, Minmin Wang, Meng Li, Qingyu Kong, Haizhao Zhao, Cuifen Zhao

**Affiliations:** Department of Pediatrics, Qilu Hospital of Shandong University, Jinan, China

**Keywords:** children, catecholamines, electrolytes, urine test, orthostatic intolerance, prediction of recurrence

## Abstract

**Background:**

Orthostatic intolerance (OI) is usually mediated by the autonomic nerve and most often happens in the upright position. However, it can also occur in other positions and can be relieved by lying down while likely to have another attack after relief. In the current study, we aim to evaluate the predictive effect of catecholamines and electrolytes on the recurrence of OI in children.

**Materials and methods:**

Children who were diagnosed with vasovagal syncope (VVS), postural tachycardia syndrome (POTS), and VVS combined with POTS were enrolled in this retrospective study and were followed up after 1-year physical treatment. Catecholamines in urine collected within 24 h, renin, angiotensin II, aldosterone in plasma, and electrolytes in both blood and urine collected in the morning were tested. A multivariate analysis and a receiver operating characteristic curve were used to validate the prediction effect.

**Results:**

In the VVS cohort, the 24 h urine adrenaline (AD) and norepinephrine (NE) levels of the non-recurrence group were lower than the 24 h urine AD and NE levels of the recurrence group, with a significant difference of *P* < 0.05. A different content can also be witnessed in the POTS cohort that the urine of the non-recurrence group contained lower sodium and chlorine. As for the VVS + POTS cohort, the non-recurrence group has lower AD and NE levels and higher potassium and phosphorus levels in urine, the difference of which proved prominent as well.

**Conclusion:**

The study provides further evidence that AD, NE, and electrolytes in urine are promising factors that are closely related to the recurrence of OI in children. The integrated evaluation system merging AD and NE may have better predictive ability.

## Introduction

Orthostatic intolerance (OI) is a common condition in the pediatric population, which presents various symptoms while standing, such as lightheadedness, blurred vision, palpitations, tremor, fatigue, and even syncope, and is relieved after recumbency (1). It encompasses vasovagal syncope (VVS), postural tachycardia syndrome (POTS), orthostatic hypotension (OH), orthostatic hypertension (OHT), and other types, among which VVS and POTS are the most prevalent (2). The widely accepted doctrine of the underlying causes of VVS and POTS is neural-mediated syncope, which refers to syncope associated with autonomic neural reflexes or autonomic nerve dysfunction and accounts for 75% of pediatric syncope cases (3). A study observed that dynamic changes in neurohormones, such as vasopressin, endothelin-1, adrenomedullin, brain and atrial natriuretic peptide (BNP and ANP), and serotonin, occurred during VVS events in adult patients (4). Other etiologies of POTS include abnormally increased sympathetic activity, excess circulating catecholamines (CAs), and absolute hypovolemia due to low blood volume. Normally, standing results in a shift of blood from the chest to the lower abdomen and legs, as well as a transfer of plasma volume from the vasculature into the interstitial space, which reduces venous return and causes a transient decrease in cardiac filling, stroke volume, and arterial pressure (5). Unloading of baroreceptors triggers compensatory sympathetic activation, which increases heart rate and leads to systemic vasoconstriction. This compensation results in the restoration of venous return and cardiac output. Neuroendocrine responses are activated, such as an increase in the release of arginine vasopressin, a decrease in the secretion of the atrial natriuretic peptide, and the activation of the renin–angiotensin–aldosterone system (RAAS). CA is a type of nerve substance composed of both catechol and amine compounds. It is mainly secreted by the adrenal medulla, extrarenal chromaffin bodies, and adrenal neurons. The main types of CA include adrenaline (AD, also known as epinephrine, Epi), norepinephrine (NE), and dopamine (DA). In patients with VVS, sudden changes in body position can cause venous congestion and a rapid decrease in left ventricular blood volume. In turn, this can trigger the Bezold–Jarisch reflex, leading to an increase in sympathetic tension and a strong release of CAs (6). Fu et al. (7) showed that the imbalance of NE and AD, with higher circulating AD, most likely contributes to vasodilation or impairments in peripheral vascular resistance by stimulating vasodilating beta-2 adrenergic receptors in skeletal blood vessels, which results in low blood pressure/cardiac output in OH and syncope subjects.

With respect to the treatment, a series of studies from a quarter-century ago indicated that salt supplementation can improve plasma volume and orthostatic tolerance in patients with unexplained syncope, as well as increase baroreceptor sensitivity. Garland et al. (8) demonstrated that high-sodium diets led to smaller increases in heart rate and lower NE, plasma renin activity, aldosterone, and RAAS activity while upright than low-sodium diets. Symptom severity in POTS patients has been demonstrated with a clear association between 24 h urinary sodium (U-Na) excretion and severity level. Moreover, symptom improvement has been linked to initial sodium content, and patients that positively responded to salt supplementation typically exhibit low 24 h U-Na excretion (9). The recurrent or sustained symptoms of OI, such as frequent syncope episodes or chronic systemic symptoms, can have negative effects on children's physical and mental health, ultimately lowering the quality of life for the whole family (10). In a retrospective study including 157 VVS patients, it was found that age may be a reliable predictor of recurrent syncope, particularly in elderly patients with positive head-up tilt test (HUTT) results. The recurrence rate of syncope increased with age, especially in women (11). Tao et al. (12) analyzed 42 children with VVS and determined that a higher number of syncope episodes before treatment, as well as a greater count of previous syncopal episodes, plays a significant role in predicting the recurrence of syncope. A compelling evidence that plasma levels of epinephrine and vasopressin increase early during HUTT and predict later decreases in blood pressure and syncope is found. Baseline and orthostatic levels of adrenomedullin, endothelin-1, and atrial natriuretic peptide, or their stable metabolites, have also been linked with susceptibility to VVS (13).

However, the predictive value of catecholamines and electrolytes in predicting the recurrence rate of OI in children remains inconclusive due to insufficient evidence. In our study, we compared the levels of catecholamines and electrolytes with both blood and urine samples in the recurrent and non-recurrence groups to identify the possible predictors contributing to the recurrence of OI in children.

## Materials and methods

### Patient enrollment and selection

The eligible candidates for the study were patients seeking medical advice at Qilu Hospital of Shandong University between January 2016 and January 2022. Further selection criteria included satisfying the following requirements: the age of the first attack (≤18 years old); experiencing syncope or premonition of syncope more than once; no presence of other diseases such as organic cardiorespiratory, neurogenic, immune, metabolic, and endocrine diseases with the etiology clearly excluded; diagnosed with OI in compliance with the diagnostic criteria in the pediatric syncope guidelines (3, 14); physical therapy as the only treatment option after discharge, including adequate physical exercise, trigger avoidance, early identification of syncope precursors, timely physical stress reduction actions, and increased intake of water and salt; and having complete medical data with no missing follow-up 1 year after physical therapy. Children who meet the above requirements will be included in the study and classified as either the recurrence group or non-recurrence group based on their 1-year physical treatment follow-up. This study was approved by the ethics committee of Qilu Hospital of Shandong University, and all participants’ guardians were fully informed of the purpose and methods of the study.

### The process of HUTT

The procedure of HUTT is directed by the current guideline (15–17). Before the experiment, drugs that may affect autonomic nervous function should be discontinued for at least five half-lives, and eating and drinking should be avoided for at least 4 h. The examination should be scheduled between 8 a.m. and 11 a.m. and equipped with a comprehensive rescue ability. Baseline and changes in blood pressure, heart rate, and electrocardiogram should be monitored and recorded throughout the experiment.

The experimental process includes the baseline head-up tilt test (BHUTT) and sublingual nitroglycerin–promoted head-up tilt test (SNHUTT). Patients lie quietly flat on the examination bed with ankle and knee joint straps fixed for 10–30 min. Then, they stand with their heads high and feet low on the test bed and tilt for 60° until the entire test is completed (45 min). If a positive reaction occurs during this process, the test needs to be terminated. If the response of the BHUTT is negative, SNHUTT can be initiated. The patient maintains the above posture, with nitroglycerin tablets (4–6 g/kg, max dose is 300 μg) under the tongue. The test should be terminated after a positive reaction occurs. If no positive reaction occurs, observation is necessary until 20 min after giving the drug.

### Collection of urine and blood samples

Venous blood samples and 24 h urine samples were taken from all enrolled children from the next day after HUTT. Blood is drawn on an empty stomach for 6 h. The urine collection procedure was as follows. Patients collected all urine from 7 o’clock in the morning until 7 o’clock the next morning in a clean large container. Responsible medical workers evenly mixed the sample, measured the total urine volume with a measuring cup, and recorded it on the test sheet. They took about 3–5 ml of the mixed urine samples and placed them in the test tube. The urine sampling was completed by then. The blood and urine samples were sent for a test as soon as possible. During the retention of samples, children were required to maintain ordinary activities. The children or guardians were informed and agreed for the collection of samples.

### Measurement of target molecules

The urine collected is preserved with concentrated hydrochloric acid at a dosage of 0.5–1 ml/100 ml to prevent bacterial growth. Quantitative determination of catecholamines in 24 h urine samples was based on high-performance liquid chromatography–mass spectrometry (HPLC–MS). The 24 h urine catecholamine test was assisted by Beijing Hehe Medical Laboratory, and the urine electrolyte and blood sample tests were assisted by the Laboratory Department of Qilu Hospital, Shandong University. The reliability of the test results had been repeatedly verified. Based on the blood samples, renin, angiotensin II, aldosterone, and electrolytes were measured. AD, NE, DA, and electrolytes were also estimated in urine samples.

### Statistical analysis

The counting data were represented by the number of examples and composition ratio or rate. When comparing two sets of counting data, the analysis method was based on the number of overall samples. When the number of overall sample (*n*) ≥ 40 , chi-square test were used; when *n*<40, analysis was conducted through Fisher exact probability method for four grid table data. When *n* was <40 or T was <1, the analysis was conducted through Fisher's exact probability method for four-grid table data. With regard to measurement data, normality tests and Levene tests were performed. The measurement data with normal distribution were expressed as mean with SD, and a *t*-test was used for comparison between the two groups. The data with skewed distribution were represented by the 25th–75th percentile, and a non-parametric rank-sum test was used for inter-group comparison. When conducting multivariate analysis on statistically significant single factors, a multivariate logistic regression model is used. *P* < 0.05 was the cut-off value that indicated statistical significance.

The area under the curve (AUC) is used in the receiver operating characteristic (ROC) curve to represent the predictive ability of predictive indicators. All of the statistical analysis relied on SPSS 25.0 statistical software, and GraphPad Prism 8.0.2 software was used for visualization.

## Results

### Characteristics of enrolled patients

A total of 320 patients were recruited for the study. Among them, 204 children were in the VVS cohort, consisting of 99 males and 105 females; 43 children were in the POTS cohort, consisting of 21 males and 22 females; and 73 children were diagnosed with VVS combined with POTS, which belonged to the VVS + POTS cohort, consisting of 31 males and 42 females. Only one female was diagnosed with OH, and no OHT child was found during this period, thus was excluded. The age range of the included children was 5–18 years old. No difference had been witnessed in the basic information, such as age, gender, and volume of 24 h urine, between the recurrence and non-recurrence groups. According to the published article, four previous syncopal episodes were recognized as a predictive indicator of syncopal recurrence-free survival rate (12). In our research, previous syncopal episodes did not exhibit a difference. Of all these episodes, the most common triggers contain long-time standing, after exercise, flag-raising ceremony, after pain, strong emotion, during class, and after postural changes. The basic information is listed in Table 1. All the tested data were found conforming to the normal distribution after normality testing.

**Table 1:** Clinical manifestation of enrolled children.

Characteristics	VVS			POTS			VVS + POTS		
	Recurrence	Non-recurrence	*P*-value	Recurrence	Non-recurrence	*P*-value	Recurrence	Non-recurrence	*P*-value
Number	43 (21%)	161 (79%)		14 (32.5%)	29 (67.5%)		18 (24.7%)	55 (75.3%)	
Age (years)	11.12 ± 4.99	10.50 ± 2.36	0.245	11.93 ± 2.02	11.07 ± 1.98	0.192	11.22 ± 2.37	11.09 ± 2.24	0.832
Gender (male)	19 (44.1%)	80 (49.6%)	0.521	8 (57.1%)	13 (44.83%)	0.449	8 (44.4%)	23 (41.8%)	0.937
Volume of 24 h urine (ml)	1,388.72 ± 544.17	1,467.04 ± 598.38	0.438	1,533.57 ± 722.84	1,312.24 ± 579.02	0.285	1,503.06 ± 587.09	1,372.36 ± 541.10	0.387
Number of seizure									
≤4	16 (72.7%)	78 (80.4%)		3 (75%)	6 (85.7%)		6 (85.7%)	21 (80.8%)	
>4	6 (27.3%)	19 (19.6%)		1 (25%)	1 (14.3%)		1 (14.3%)	5 (19.2%)	
Predisposition									
Total predisposition	38	122		13	9		27	11	
Long-time standing	7 (18.4%)	29 (23.8%)		1 (7.7%)	3 (33.3%)		8 (29.6%)	6 (54.5%)	
After exercise	10 (26.3%)	26 (21.3%)		4 (30.8%)	4 (44.4%)		8 (29.6%)	4 (36.4%)	
Flag-raising ceremony	1 (2.6%)	10 (8.2%)		—	—		—	—	
After pain	1 (2.6%)	9 (7.4%)		—	—		3 (11.1%)	—	
After strong emotion	2 (5.3%)	7 (5.7%)		2 (15.4%)	1 (11.1%)		5 (18.5%)	1 (9.1%)	
During class	4 (10.5%)	8 (6.6%)		2 (15.4%)	1 (11.1%)		—	—	
After postural changes	3 (7.9%)	6 (4.9%)		4 (30.8%)	—		3 (11.1%)	—	

VVS, vasovagal syncope; POTS, postural tachycardia syndrome.

### Indicators of recurrence

**Figure 1:**
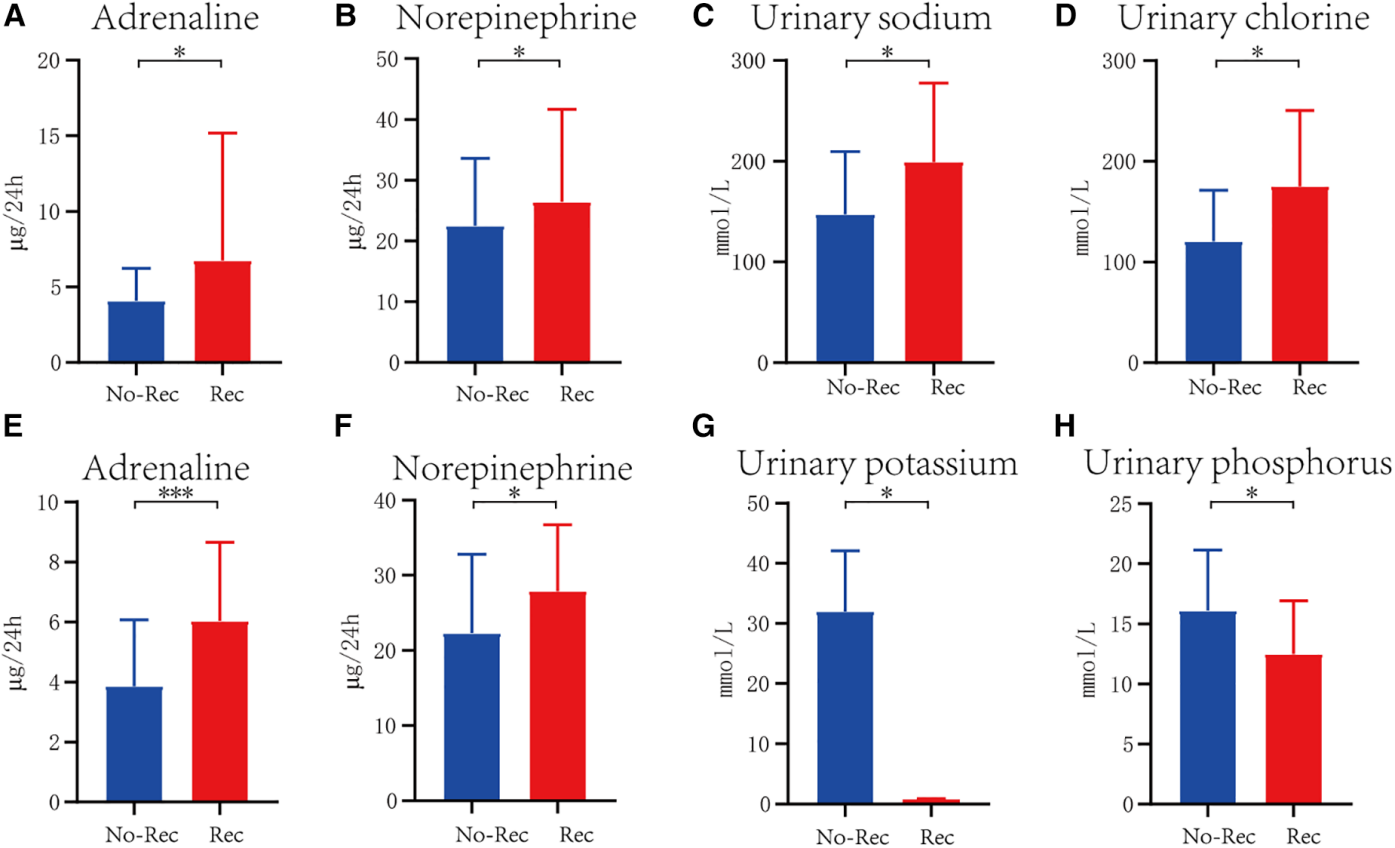
Significant difference of indicator between recurrence and non-recurrence groups in VVS cohort (**A,B**), POTS cohort (**C,D**), and VVS combined with POTS cohort (**E–H**). The blue columns represent patients without recurrence, marked as No-Rec, whereas the red columns indicate patients with recurrence. *P < .05, **P < .01, ***P < .001.

In each cohort, all the monitored indexes were compared between the recurrence group and the non-recurrence group (Table 2). Out of 204 children diagnosed with VVS, 43 had experienced recurrence, accounting for 21.8%, and 160 had non-recurrence. Urinary adrenaline (U-AD) and urinary norepinephrine (U-NE) levels were higher in the recurrence group than the U-AD and U-NE levels in the non-recurrence group, with statistical significance. Urinary electrolyte and blood electrolyte levels were equivalent in these two groups (Figures 1A,B). In the POTS cohort, significant differences in urinary potassium (U-K) (199.38 ± 78.16 vs. 147.33 ± 62.22, *P* < 0.05) and urinary phosphorus (U-P) (175.25 ± 75.19 vs. 120.52 ± 50.77, *P* < 0.05) between the two groups can be witnessed, both of which had high content in the recurrence group. The difference in other detected values was obscure (Figures 1C,D). When it comes to the VVS + POTS cohort, U-AD was prominently differently contained between the recurrence and non-recurrence groups (6.03 ± 2.63 vs. 3.75 ± 2.06), with a *P*-value of <0.001. Other indicators, such as U-NE, U-K, and U-P, also exhibited differences. Among all these dominant indicators, urinary catecholamine was higher in the recurrence group, while urinary electrolyte was higher in the non-recurrence group (Figures 1E–H). All the results above indicate that urinary catecholamine and urinary electrolytes are promising predictors in the recurrence of VVS and POTS.

**Table 2:** Evaluation and comparison of potential factors between groups.

Indicators	VVS			POTS			VVS + POTS		
	Recurrence	Non-recurrence	*P*-value	Recurrence	Non-recurrence	*P*-value	Recurrence	Non-recurrence	*P*-value
U-AD (μg/24 h)	6.75 ± 8.43	4.10 ± 2.13	0.047*	5.42 ± 2.29	5.74 ± 3.57	0.765	6.03 ± 2.63	3.75 ± 2.06	<0.001***
U-NE (μg/2 h)	26.45 ± 15.18	22.53 ± 11.09	0.049*	27.81 ± 15.48	26.12 ± 17.14	0.757	27.90 ± 8.86	22.02 ± 10.40	0.035*
U-DA (μg/24 h)	270.48 ± 108.75	242.34 ± 101.52	0.113	312.23 ± 117.21	258.35 ± 97.95	0.121	238.40 ± 63.18	239.50 ± 78.42	0.957
U-K (mmol/L)	29.04 ± 8.96	33.91 ± 14.64	0.118	42.47 ± 18.68	32.24 ± 17.66	0.181	25.94 ± 8.05	32.72 ± 9.20	0.033*
U-Na (mmol/L)	147.00 ± 59.83	152.12 ± 63.34	0.715	199.38 ± 78.16	147.33 ± 62.22	0.036*	134.09 ± 79.50	158.43 ± 61.54	0.287
U-Cl (mmol/L)	123.29 ± 54.99	128.01 ± 55.87	0.705	175.25 ± 75.19	120.52 ± 50.77	0.032*	110.64 ± 69.22	133.81 ± 53.37	0.244
U-Ca (mmol/L)	2.36 ± 1.50	2.24 ± 1.51	0.718	2.93 ± 1.20	2.85 ± 1.51	0.888	1.96 ± 1.18	2.54 ± 1.60	0.27
U-P (mmol/L)	15.75 ± 5.16	15.06 ± 5.58	0.575	18.41 ± 9.07	14.79 ± 6.80	0.253	12.48 ± 4.42	16.26 ± 5.03	0.030*
Plasma renin (pg/ml)	28.22 ± 36.99	17.30 ± 15.11	0.22	22.08 ± 14.59	22.12 ± 21.98	0.997	17.16 ± 10.51	19.64 ± 15.26	0.705
Plasma angiotensin II (pg/ml)	97.78 ± 33.14	127.56 ± 93.34	0.185	124.62 ± 46.87	132.05 ± 50.98	0.701	104.84 ± 28.63	127.00 ± 78.14	0.467
Plasma aldosterone (pg/ml)	182.01 ± 119.48	235.99 ± 188.11	0.231	176.54 ± 78.07	233.90 ± 164.87	0.293	136.76 ± 42.30	159.02 ± 123.39	0.667
B-K (mmol/L)	4.27 ± 0.28	4.37 ± 0.27	0.099	4.32 ± 0.17	4.34 ± 0.37	0.834	4.33 ± 0.23	4.36 ± 0.29	0.749
B-Na (mmol/L)	141.04 ± 2.31	140.87 ± 2.41	0.753	141.42 ± 1.38	140.75 ± 2.31	0.374	140.40 ± 2.46	140.77 ± 1.75	0.588
B-Cl (mmol/L)	105.88 ± 2.66	105.52 ± 2.42	0.517	106.92 ± 1.56	105.85 ± 2.39	0.179	106.30 ± 2.21	103.87 ± 17.27	0.662
B-Ca (mmol/L)	2.34 ± 0.38	2.37 ± 0.30	0.623	2.40 ± 0.06	2.33 ± 0.39	0.534	2.45 ± 0.13	2.33 ± 0.36	0.288
B-P (mmol/L)	1.64 ± 0.24	1.65 ± 0.19	0.763	1.59 ± 0.15	1.64 ± 0.13	0.423	1.61 ± 0.19	1.70 ± 0.22	0.267

VVS, vasovagal syncope; POTS, postural tachycardia syndrome; U-AD, urinary adrenaline; U-NE, urinary norepinephrine; U-DA, urinary dopamine; U-K, urinary potassium; U-Na, urinary sodium; U-Cl, urinary chlorine; U-Ca, urinary calcium; U-P, urinary phosphorus; B-K, blood potassium; B-Na, blood sodium; B-Cl, blood chlorine; B-Ca, blood calcium; B-P, blood phosphorus.

* *P*-value < 0.05.

** *P*-value < 0.01.

*** *P*-value < 0.001.

### Multi-factor regression analysis

**Figure 2:**
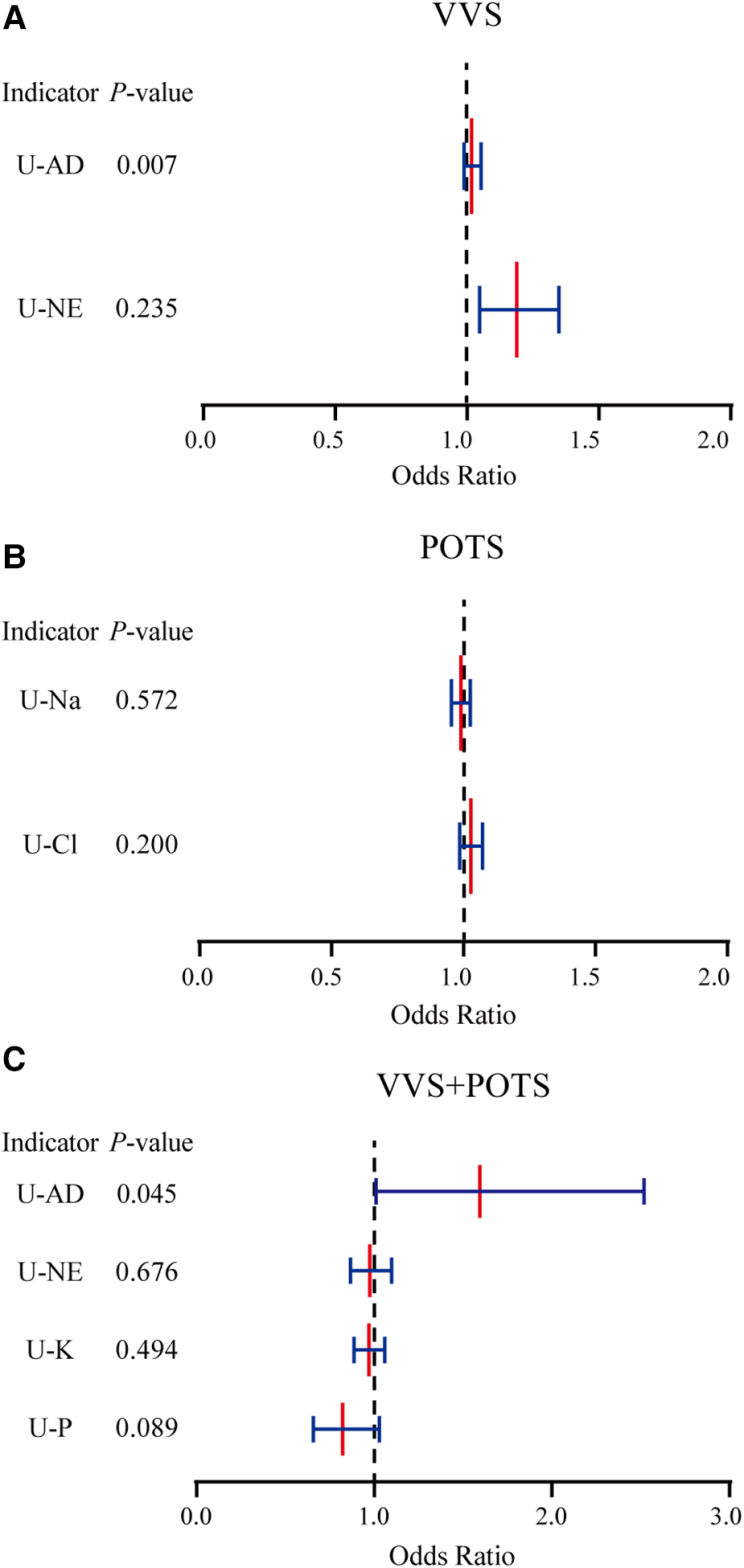
Multivariate logistic regression of the significant prognostic factors in three cohorts.

U-AD was identified as an independent prognostic factor in the VVS + POTS cohort, being the risk factor for prognosis. Other significant factors that had undergone single regression testing had been found not to be independent prognostic factors after multiple factor analysis (Figure 2 and Table 3).

**Table 3:** Multivariate analysis of indicators.

Indicators	b	S_b_	Wals	O^R	*P*	95% CI
VVS						
U-AD	0.173	0.064	7.244	1.017	0.007	0.989–1.054
U-NE	0.017	0.014	1.408	1.189	0.235	1.048–1.349
POTS						
U-Na	0.011	0.019	0.319	0.990	0.572	0.954–1.026
U-Cl	0.027	0.021	1.641	1.028	0.200	0.986–1.072
VVS + POTS						
U-AD	0.468	0.233	4.034	1.596	0.045	1.011–2.519
U-NE	−0.025	0.060	0.175	0.975	0.676	0.867–1.097
U-K	−0.031	0.469	0.469	0.970	0.494	0.887–1.059
U-P	−0.195	0.115	2.895	0.823	0.089	0.657–1.030

VVS, vasovagal syncope; POTS, postural tachycardia syndrome; U-AD, urinary adrenaline; U-NE, urinary norepinephrine; U-K, urinary potassium; U-Na, urinary sodium; U-Cl, urinary chlorine; U-P, urinary phosphorus.

### Prediction effect estimation and truncation value calculation

**Figure 3:**
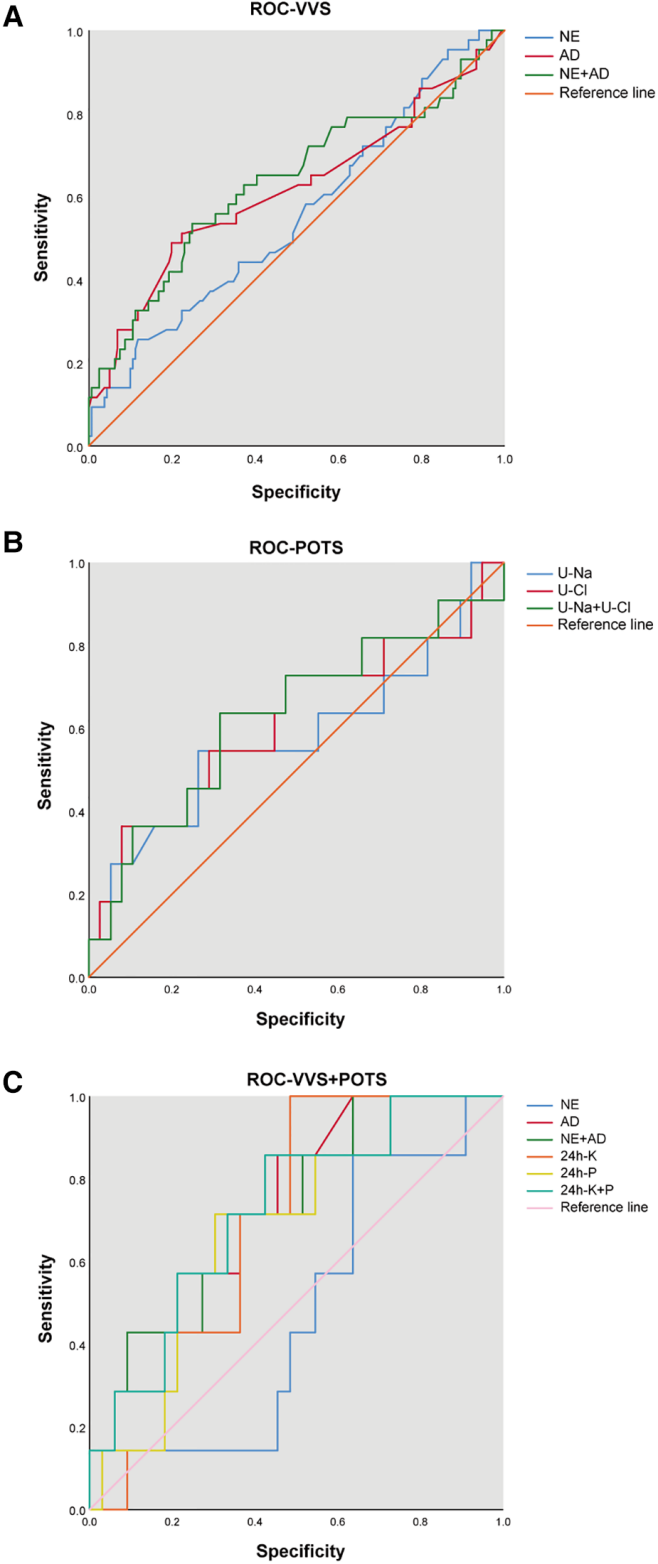
ROC curve of the promising indicator in different cohorts.

The ROC curve was utilized to estimate the prediction accuracy of the predictive factors. U-AD and U-NE in the VVS cohort and VVS + POTS cohort had great predictive effects with AUC of >0.5, while having different cut-off values. U-Na and urinary chlorine (U-Cl) in the POTS cohort exhibited good predictive effects, demonstrating the possible biochemical changes that may occur after syncope occurred (Figure 3 and Table 4).

**Table 4:** The predictive value of single and combined indicators for recurrence.

Indicators	AUC (95% CI)	*P*-value	Cut-off value	Sensitivity	Specificity	Youden index
VVS						
U-AD	0.617 (0.510–0.723)	0.019	5.62 μg/24 h	0.488	0.801	0.290
U-NE	0.557 (0.458–0.655)	0.254	33.50 μg/24 h	0.256	0.882	0.138
U-AD + U-NE	0.363 (0.260–0.466)	0.006	—	—	—	—
POTS						
U-Na	0.685 (0.453–0.916)	0.130	208.50 mmol/L	0.500	0.905	0.405
U-Cl	0.708 (0.484–0.933)	0.088	89.00 mmol/L	1.000	0.333	0.333
U-Na + U-Cl	0.280 (0.075–0.485)	0.071	—	—	—	—
VVS + POTS						
U-AD	0.738 (0.577–0.899)	0.130	5.53 μg/24 h	0.545	0.414	0.405
U-NE	0.612 (0.429–0.796)	0.260	16.74 μg/24 h	0.909	0.368	0.278
U-K	0.297 (0.150–0.443)	0.042	—	—	—	—
U-P	0.318 (0.155–0.482)	0.069	—	—	—	—
U-AD + U-NE	0.237 (0.096–0.378)	0.008	—	—	—	—
U-K + U-P	0.280 (0.134–0.425)	0.027	—	—	—	—

VVS, vasovagal syncope; POTS, postural tachycardia syndrome; U-AD, urinary adrenaline; U-NE, urinary norepinephrine; U-K, urinary potassium; U-Na, urinary sodium; U-Cl, urinary chlorine; U-P, urinary phosphorus.

## Discussion

### VVS group

The present study reveals elevated levels of 24 h U-AD and U-NE in the recurrence group, indicating their potential value as predictive markers for VVS recurrence. Benditt et al. (18) previously reported an increase in the AD/NE ratio among VVS patients during stable hemodynamics following HUTT, suggesting that alterations in CA levels may play a crucial role in VVS triggers instead of being secondary to hemodynamic fluctuations. A recent study involving 827 patients has established a correlation between a higher orthostatic AD level on the tilt posture and a shorter time to the onset of the VVS reflex. During orthostasis, a greater increase in AD and vasopressin was indicative of an imminent onset of VVS (19). In our study, the logistic multivariate regression analysis indicated that the high U-NE level was an independent risk factor for VVS recurrence in children. Jardine et al. (20) conducted an investigation involving prolonged HUTT at 60°. The study revealed that NE and AD levels increased early during upright posture to a greater extent in fainters than controls. However, in the proximity of syncope, AD levels continued to increase, while NE levels fell back to control values. In our study, the AUC of U-AD was higher than the AUC of U-NE in the ROC curve (0.617 vs. 0.557). This finding implies that U-AD exhibits a higher predictive value than U-NE.

The optimal cut-off value of U-AD for predicting VVS recurrence was 5.62 µg/24 h, with a sensitivity of 0.488 and a specificity of 0.801. Therefore, a 24 h AD level exceeding 5.62 µg indicates a greater likelihood of VVS recurrence, showing a high CA state in the children of the recurrence group. During the onset of decompression reflex and pressure, the human heart experiences excessive contractions, stimulating the baroreceptors in the posterior lower wall of the left ventricle, which can trigger the Bezold–Jarisch reflex and lead to syncope in children. Moreover, several studies have discovered that females diagnosed with VVS exhibit lower autonomic activity than males, particularly in terms of sympathetic activity. This is the possible reason for the higher urinary noradrenaline concentration in the male group than the urinary noradrenaline concentration in the female group (*P* < 0.05), as well as the higher U-AD concentration in the non-recurrence group (*P* < 0.01).

A previous exploration of the management of pediatric VVS highlights the significance of health education, increased salt and water intake, pharmacological treatment, and pacemaker intervention (21). Theoretically, increasing salt and water intake can not only directly increase plasma volume but also influence the regulation of cerebral and peripheral vessels during standing, ultimately improving the individual's orthostatic tolerance (22). Furthermore, increasing salt and water intake has been linked to a decline in sympathetic activity in the positive pressure state, which diminishes the likelihood of triggering the Bezold–Jarisch reflex and ultimately reduces syncope occurrences in VVS patients (23). Wang et al. conducted a meta-analysis of five randomized controlled trials, which demonstrated that increasing salt and water intake was more effective than significantly improving the response observed in HUTT and reducing the recurrence of syncope in the control group (24). In our study, it was observed that children in the recurrence group exhibited lower U-Na and 24 h urine volume than the non-recurrence group, albeit without statistical significance. Consequently, children should be administered water and ORS supplements if their 24 h urine volume and sodium levels are lower than the normal standards in order to minimize the likelihood of recurrence in the future.

### POTS group

In an expert consensus statement issued in 2015 by the Heart Rhythm Society, POTS was defined as a condition characterized by a recurrent increase of heart rate by 30 beats per minute while upright without the presence of OH. This syndrome occurs when the peripheral vasculature fails to maintain adequate constriction to allow excessive venous pooling during upright posture (25). Our study reveals a statistical difference in 24 h U-Na and chlorine levels between the two groups (*P* = 0.036; *P* = 0.032). The ROC curve demonstrated a sensitivity of 50% and specificity of 90.5% for the correct prediction of responders. According to the 2020 to 2025 Dietary Guidelines for Americans, it is recommended to consume less than 100 mEq sodium per day. The average daily sodium intake in the United States is reported to be 137 mEq for women and 186 mEq for men. Increased salt intake is a cornerstone recommendation in the management of OH in clinical guidelines, including a Class I recommendation in the European Society of Cardiology (ESC)—Treatment of Syncope: Orthostatic Hypotension, 2018 (26). In a recent study, patients with POTS experienced decreases in the increase of heart rate, supine and upright heart rate, and standing plasma NE after a short-term period of dietary sodium intake of 300 mEq/day, in comparison with a period of dietary sodium intake of 10 mEq/day (8). The cut-off value of U-Na predicting POTS was 208.5 mmol/L.

### VVS combined with POTS group

Autonomic nerve disorders and elevated catecholamine levels are the dominant pathogeneses in children experiencing OI. VVS commonly manifests with heightened vagal tone as a result of transient sympathetic excessive excitation. Some children with POTS display amplified sympathetic tone, while others demonstrate reduced sympathetic activity. The latter form of POTS, known as hyperadrenergic POTS, constitutes approximately 10% of all POTS cases (27). Goldstein et al. noted that patients with POTS experience sympathetic activation in the supine position and increased adrenal medulla hormones contribute to relative tachycardia. In contrast, no such activation occurs in individuals with neurocardiogenic syncope (28). Notably, in our study, the levels of U-AD (*P* < 0.001) and U-NE (*P* < 0.05) were higher in the recurrence group. Furthermore, the multivariate analysis revealed that elevated urinary epinephrine was identified as a risk factor associated with recurrence in VVS children with POTS. Another study found that patients with hyperadrenergic POTS may exhibit a centrally mediated drive of NE or a defect in NE reuptake, leading to the increased availability of NE at synaptic junctions (29). In our study, a ROC curve was utilized to analyze the respective predictive values of U-AD and U-NE, which revealed that urinary EPI had a higher predictive value than NE (AUC: 0.738 vs. 0.612). The optimal cut-off value for U-AD in predicting VVS + POTS was 5.53 µg/24 h, with a sensitivity of 0.545 and a specificity of 0.868.

The 2018 ESC Guidelines for the diagnosis and management of syncope recommend adequate salt and water intake for patients without hypertension, with a target of 2–3 L of fluids per day and 10 g of sodium chloride. Rapid consumption of cool water has been proven effective in combating postprandial hypotension and OI (16). Several regimens suggest increasing salt intake for patients with recurrent syncope based on the typical daily intake of the general population, which equates to 3,400 mg of sodium per day, 8.5 g of sodium chloride, or 150 mmol of sodium chloride per day. Zhang et al. reported that POTS children exhibited lower levels of 24 h U-Na than healthy children and the U-Na level was negatively correlated with clinical symptom severity. When 24 h U-Na was at 124 mmol, the sensitivity and specificity for identifying POTS children were 76.9% and 93%, respectively (9). Cui et al. discovered statistically significant differences in the treatment methods of children with POTS, VVS, and VVS associated with POTS. Notably, a greater number of children received drug treatment due to the more severe clinical symptoms observed in patients with POTS and VVS (*P* < 0.01) (30). In this study, the U-Na and U-Cl levels in male children were significantly higher than the U-Na and U-Cl levels in female children (*P* < 0.01). Furthermore, the levels of U-Na and urinary chloride in the female group were significantly lower than the levels of U-Na and urinary chloride in the recurrence group (*P* < 0.05). These results imply that early or combined drug therapy may be beneficial for children, especially female children, with low U-Na levels to reduce symptoms and prevent recurrence.

Urinary catecholamines and electrolytes are readily obtainable and less invasive compared with other diagnostic indicators. Our findings offer indirect validation of the significance of neurohumoral hormones in the pathophysiological mechanism of OI in children. Subsequently, conducting further research to analyze disease treatment and prognostic measures based on catecholamines holds the potential to optimize patient management by healthcare professionals. Oner et al. proposed that the deficiency of vitamin B12 may result in sympathetic nervous system baroreceptor dysfunction among patients with POTS. Furthermore, due to the impairment of enzymes reliant on vitamin B12, namely, phenylethanolamine N-methyltransferase and catecholamine-O-methyltransferase, these enzymes are involved in the conversion and degradation of noradrenaline, resulting in significantly elevated blood levels of noradrenaline (31). In addition, the assessment of urinary catecholamines in first-time onset children can facilitate proactive doctor intervention, which typically involves early drug intervention.

Still, limitations in the present study exist. This study is a retrospective study conducted at a single center, and it still has some limitations. First, this study is a single-center retrospective study. Therefore, further investigation through large sample and multicenter studies is necessary to determine the generalizability of the results to children and adolescents in different geographical areas. Second, it is crucial to acknowledge that all medical history data utilized in this study were obtained from the patients, their family members, or witnesses, which introduces the possibility of recall bias in subjective descriptions. Despite the above limitations, our research still proved novel evidence for the prediction of the recurrence.

## Conclusion

Our study demonstrated a significant difference in 24 h urine AD levels among children with recurrent VVS and POTS. The cut-off value for AD prediction is 5.53 µg/24 h. This finding suggests that the 24 h urine AD levels could be of great help in the individual management strategy of OI in children and adolescents.

## Data Availability

The raw data supporting the conclusions of this article will be made available by the authors, without undue reservation.
